# ESPO AND HEALTH SCIENCES SECTION SYMPOSIUM: STRONGER TOGETHER: INTEGRATING CARE PARTNERS AND SOCIAL SUPPORT TO IMPROVE HEALTH OUTCOMES

**DOI:** 10.1093/geroni/igae098.0761

**Published:** 2024-12-31

**Authors:** Katherine Britt, Kate Perepezko, Beth Fields

**Affiliations:** Chair; Co-Chair; Discussant

## Abstract

Millions of care partners (relatives, partners, or other support persons) provide support to older adults; however, they are not consistently included in health care and social service delivery. This omission is detrimental as care partner inclusion has been shown to improve older adult outcomes, including symptom management, functioning, and mental health (Weinberg et al., 2007). To highlight how care partners can be involved in services and interventions, this symposium will present innovative approaches to integrate care partners and social support to improve health outcomes for older adults. This session highlights the work of early career scientists whose research examines how care partners can be included in interventions to support the health of the older adult for whom they provide care and for themselves. Dr. Mary Wyman will present her work exploring associations of caregiving characteristics (e.g., amount received, number of caregivers, any formal help on board, and primary caregiver gender & relationship) with the utilization of mental health services among older veterans. Dr. DeAnnah Byrd will present on satisfaction with social support, stress, and cognition in older Black Adults. Dr. Katherine Britt will present a qualitative study with providers on the role of family caregivers in cognitive care planning. Dr. Kate Perepezko will present preliminary findings from the Community Aging in Place Advancing Better Living for Elders (CAPABLE): A Scale-Up Project, characterizing care partners’ involvement in an aging-in-place intervention. This symposium will conclude with a discussion led by Dr. Beth Fields that will connect each of the presentations.

